# FMCA-DTI: a fragment-oriented method based on a multihead cross attention mechanism to improve drug–target interaction prediction

**DOI:** 10.1093/bioinformatics/btae347

**Published:** 2024-05-29

**Authors:** Qi Zhang, Le Zuo, Ying Ren, Siyuan Wang, Wenfa Wang, Lerong Ma, Jing Zhang, Bisheng Xia

**Affiliations:** College of Mathematics and Computer Science, Yan'an University, Yan'an 716000, China; College of Mathematics and Computer Science, Yan'an University, Yan'an 716000, China; College of Mathematics and Computer Science, Yan'an University, Yan'an 716000, China; College of Mathematics and Computer Science, Yan'an University, Yan'an 716000, China; College of Mathematics and Computer Science, Yan'an University, Yan'an 716000, China; College of Mathematics and Computer Science, Yan'an University, Yan'an 716000, China; Medical College of Yan'an University, Yan'an University, Yan'an 716000, China; Medical Research and Experimental Center, The Second Affiliated Hospital of Xi'an Medical University, Xi'an 710021, China; College of Mathematics and Computer Science, Yan'an University, Yan'an 716000, China

## Abstract

**Motivation:**

Identifying drug–target interactions (DTI) is crucial in drug discovery. Fragments are less complex and can accurately characterize local features, which is important in DTI prediction. Recently, deep learning (DL)-based methods predict DTI more efficiently. However, two challenges remain in existing DL-based methods: (i) some methods directly encode drugs and proteins into integers, ignoring the substructure representation; (ii) some methods learn the features of the drugs and proteins separately instead of considering their interactions.

**Results:**

In this article, we propose a fragment-oriented method based on a multihead cross attention mechanism for predicting DTI, named FMCA-DTI. FMCA-DTI obtains multiple types of fragments of drugs and proteins by branch chain mining and category fragment mining. Importantly, FMCA-DTI utilizes the shared-weight-based multihead cross attention mechanism to learn the complex interaction features between different fragments. Experiments on three benchmark datasets show that FMCA-DTI achieves significantly improved performance by comparing it with four state-of-the-art baselines.

**Availability and implementation:**

The code for this workflow is available at: https://github.com/jacky102022/FMCA-DTI.

## 1 Introduction

Drug discovery and drug repositioning are valuable research areas in biomedicine, and determining drug–target interactions is essential for drug discovery and drug repositioning, promoting understanding of drug mechanisms of action, disease pathology, and drug side effects (Agamah *et al.* 2020, Luo *et al.* 2021). Predicting drug–target interactions through traditional biomedical *in vitro* experiments is reliable, but time-consuming, expensive, and challenging to implement on large-scale data (Whitebread *et al.* 2005, Thafar *et al.* 2020, Bai *et al.* 2023). Statistically, developing a new drug costs about 1.8 billion dollars and lasts 13 years (Whitebread *et al.* 2005, Hopkins 2009, Mahmud *et al.* 2021). In addition, numerous drug–target interactions remain undiscovered in the compound and bioactivity databases. For instance, the US Food and Drug Administration (FDA) has only approved roughly 2110 small molecule drugs and 4964 experimental drugs (Chen *et al.* 2016). DrugBank (Wishart *et al.* 2006) shows that only 3150 are associated with these drugs among approximately 20 000 human proteins.

With the continuous accumulation of large amounts of genomic, biochemical and biomedical data and the rapid development of computing power, computational approaches have become one of the most important techniques for identifying DTI in drug discovery. The search scope for candidate compounds can be significantly narrowed by computer simulation methods, leading to reducing research costs, accelerating drug development and providing insights into the causes of potential side effects in drug combinations (Yao *et al.* 2010, Ezzat *et al.* 2016, Lim *et al.* 2019). In recent years, many computational methods have been proposed for predicting drug–target interactions, and these methods can be summarized into three categories: ligand-based methods (Keiser *et al.* 2007), structure-based methods (Cheng *et al.* 2007, 2012, Morris *et al.* 2009, Zitnik *et al.* 2018), and machine learning-based approaches (He *et al.* 2010, Liu *et al.* 2011, Li and Cai 2019, Zhang *et al.* 2019, Peng *et al.* 2020, Bagherian *et al.* 2021, Shang *et al.* 2021). Ligand-based approaches rely on the assumption that ligands with similar chemical properties have similar biological activities and bind to similar target proteins (Napolitano *et al.* 2013). Such approaches rely on a priori knowledge of the biologically active ligands and the structure of proteins, and predict the ligand–protein interactions by utilizing the structural similarities between the most chemically related proteins (Liu *et al.* 2013). However, the results of these methods may become unreliable when the number of ligands known to bind to the target protein is insufficient. Structure-based methods utilize 3D substructures of compounds and proteins and dynamic simulations to determine DTI (Cheng *et al.* 2007), such as molecular docking, molecular dynamics simulations, and binding free energy predictions. However, these methods are overly dependent on the 3D structure of the protein, and the accuracy of the prediction decreases when the 3D structural information of the protein is unknown.

A machine learning-based approach translates drugs and proteins and proven drug–target interactions into feature vectors to train predictive models (Bagherian *et al.* 2021). Deep learning in machine learning has achieved excellent performance in natural language processing and image recognition, and it is now widely used in bioinformatics. Many deep learning-based methods of DTI prediction have also been proposed with competitive results. These methods mainly consist of two steps: feature extraction and interaction prediction (Bagherian *et al.* 2021). Usually, three attributes of the drug (biological, topological, and physicochemical information), as well as target information, are generated as feature vectors or matrices (Yamanishi *et al.* 2008). These methods represent drug–protein pairs as fixed-length feature vectors that reflect the physical, chemical, and molecular properties (Yamanishi *et al.* 2008, Bagherian *et al.* 2021).

Deep learning methods view the DTI prediction task as a binary classification problem, where the positive class consists of interacting drug–target pairs and the negative class consists of noninteracting drug–target pairs (Faulon *et al.* 2008), respectively represented by binary labels 1 and 0. Convolutional neural networks (CNNs) have translational invariance and can handle high-dimensional features (Peng *et al.* 2020), resulting in many methods using CNNs to predict DTI. For example, DeepConv-DTI (Lee *et al.* 2019) used a fully connected network and a 1D CNN to, respectively, extract the extended connectivity fingerprint of a drug and the amino acid sequence of a protein features, and predicted the results via a concatenation operation and a fully connected layer. DeepDTA (Öztürk *et al.* 2018) used CNNs to extract low-dimensional features of compounds and protein sequences; then, the resulting feature vectors are fed into the fully connected layer to compute the final predictions. HyperAttentionDTI (Zhao *et al.* 2022) proposed a model based on CNNs and a hyperattention mechanism. Two CNN blocks are used to extract drug and protein features, hyperattention is used to capture their interaction information, and the fully connected layer realizes the prediction. Some approaches use a combination of multiple deep learning models, such as DeepEmbedding-DTI (Chen *et al.* 2021), which proposed a graph neural network with bidirectional long-short-term memory (BiLSTM) of attention to predict DTI. For efficient training, a bidirectional encoder of the transformer was used to extract substructure features from protein sequences, and a local breadth-first search was used to learn the subgraph information from the molecular graph. MolTrans (Huang *et al.* 2021) encoded the drug and protein sequences using the transformer to get enhanced drug and protein feature embeddings, then the interaction matrix of drug and protein is obtained by inner product operation, and finally, the prediction was carried out with CNNs and a fully connected neural network.

Fragments (substructures in the structure of a drug) play a key role in drug activity. Many studies (Rogers and Hahn 2010, Maas *et al.* 2013, Laufkötter *et al.* 2019, Arús-Pous *et al.* 2020, Dou *et al.* 2023) have introduced several methods for mining drug substructures: the extended-connectivity fingerprints (ECFPs) is a common method for encoding molecular substructures (Rogers and Hahn 2010), as they consist of a series of binary integers, where the presence or absence of a specific substructure in a molecule is indicated by a 1 or 0 on each molecule (Probst and Reymond 2018, Laufkötter *et al.* 2019). Byte pair encoding (BPE) is a data-driven segmentation approach that splits simplified molecular input line entry system (SMILES) into fragments without relying on any domain knowledge (Arús-Pous *et al.* 2020), where SMILES, proposed by David Weininger, represents molecules using the concept of graphs with atoms as nodes and edges as bonds (Weininger 1988). The retrosynthetic combinatorial analysis procedure (RECAP) fragments molecules electronically based on chemical knowledge (Lewell *et al.* 1998). BCM-DTI (Dou *et al.* 2023) proposed two different segmentation strategies. One is branch chain mining (BCM) for drug segmentation, which considers the branch chain a specific drug fragment. BCM segments SMILES into three types of fragments, including the branch chain, common substructures, and fragments, aiming to enhance the functionality and diversity of the fragments. The other is the category fragment mining (CFM) approach for protein, where protein sequences are first mapped into labeling categories from A to H and then segmented into different amino acid fragments using K-gram. After the mentioned processes, two CNN blocks were used to learn the features of these different fragment types. However, this method only uses CNN to learn the respective features and ignores the interaction features between drug molecules and proteins.

Inspired by BCM-DTI (Dou *et al.* 2023) and previous studies (Öztürk *et al.* 2018, Peng *et al.* 2020, Zhao *et al.* 2022, Bian *et al.* 2023), we propose a fragment-oriented model based on multihead cross attention for predicting DTI, namely FMCA-DTI. FMCA-DTI is an end-to-end deep learning framework with two types of fragment segmentation methods, and the multihead cross attention mechanism for learning interaction features between different fragments. By segmenting the input sequence into different fragments, the model can extract richer information from these substructures. Additionally, in contrast to the traditional self-attention mechanism, which focuses only on the input sequence itself, the cross attention mechanism can focus on the relationships between different fragments of drugs and proteins, thus effectively enhancing the feature representation. Specifically, FMCA-DTI first obtains the embedding information of drug fragments and protein fragments through BCM and CFM. Then, learns the low-dimensional features of these fragments through two parallel CNN blocks. Next, in the shared-weight-based multihead cross attention mechanism, when computing the drug feature, we use the drug fragment features as queries and the protein fragment features as keys and values, and calculate the protein feature using protein fragment features as queries and the drug fragment features as keys and values. Finally, we use a fully connected (FCN) layer to make a DTI prediction. Experiments on three public datasets show that our method achieves excellent performance compared to state-of-the-art methods.

## 2 Materials and methods

**Figure 1.**
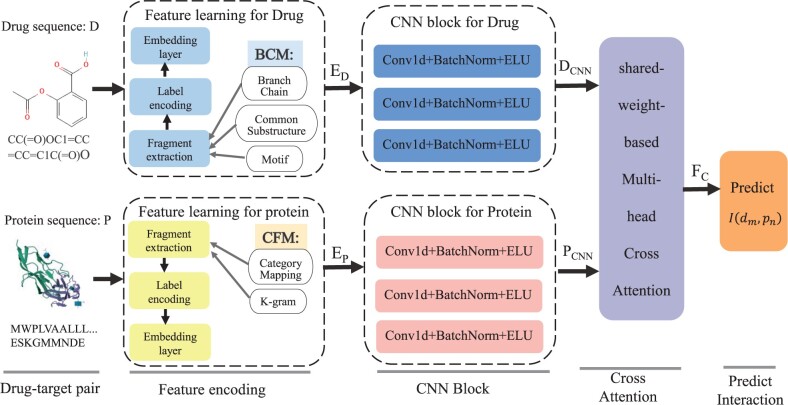
Overview of FMCA-DTI framework. The model first obtains the embedding vectors by encoding the input sequences and extracts the features of drug fragments and protein fragments by CNN. The framework, then, extracts the interaction features between the two fragments by a shared-weight-based multihead cross attention layer and implements the prediction using the interaction prediction block.

This section introduces the problem formulation and modeling framework for DTI prediction. The modeling framework is shown in Fig. 1. It consists of four parts: two feature encoding modules for mapping the fragment structure information of drugs and proteins to the embedding feature vectors; two CNN blocks for feature extraction to generate the feature matrices; a shared-weight-based multihead cross attention block is used to extract the interaction features between drug fragments and protein fragments; and a predictor for classification. In the feature encoding module, under a given drug–target pair, the various fragment types of drugs are first extracted by BCM, while the functional fragments of proteins are extracted using CFM, then, the fragment information is encoded and fed into the embedding layer to output the embedding feature vectors. In the CNN block, the embedding feature vectors act as input to the corresponding CNN block to generate feature representations; the shared-weight-based multihead cross attention layer takes the feature representations as input to extract the interaction features between the drug fragments and the protein fragments; and finally, the predictor applies the interaction feature to predict.

### 2.1 Problem formulation

Drug target interaction prediction is viewed as a binary classification task predicting whether a drug interacts with its target. Drugs are represented by SMILES (Weininger 1988) string, and now a set of M drugs $D=\left\{d_{1},d_{2},\ldots ,d_{M}\right\}$ is given. Proteins are represented by linear sequences of amino acids, a set of N proteins $P=\left\{p_{1},p_{2},\ldots ,p_{N}\right\}$ is given, and our goal is to predict whether there is an interaction (I) between $d_{m}$ and $p_{n}$:

(1) $$I\left(d_{m},p_{n}\right)=\{\begin{array}{c} 1,\mathrm{yes}\\ 0,no \end{array}$$

### 2.2 Feature encoding

As Fig. 1 shows, the feature encoding section contains three sub-layers: fragment extraction layer, label encoding layer, and embedding layer. For the feature encoding module of drugs, the first step involves BCM identifying the branch chain via the fragment extraction layer as the first type of fragment based on the generative rule that SMILES surrounded by parentheses constitute the branch chain structure. After removing all contained brackets and branch chains, the remaining fragments are spliced as the main chain, and if substructures, namely benzene, oxygen chain, and carboxyl group, are detected, they are extracted as the second type of fragment for each branch chain. Finally, the module searches for synthetic fragments that cannot be further split as the third type of fragment. Amino acids are first classified into eight categories via CFM in proteins based on their chemical properties and mapped to the corresponding A-H categories by “category mapping.” The mapped amino acid chains are then divided by K-gram, each set of amino acids consisting of three DNA bases.

### 2.3 CNN block

CNN can learn features by moving a kernel of fixed size over image or sequence data, capable of capturing local dependencies between different locations. With multilayer convolution, the feature representations can be extracted and integrated layer by layer, and the CNN-based model can perform parallel computing efficiently, significantly reducing the training time. Our proposed model involves two CNN blocks to extract fragment features of drugs and proteins. Figure 1 of the CNN block section represents these two CNN blocks and the three sublayers of each block. Each CNN block contains three convolutional layers, each consisting of a 1D convolutional layer, a batch normalization layer, and an activation function layer of exponential linear unit (ELU) (Clevert *et al.* 2015). The activation function of ELU does not encounter the problem of exploding or vanishing gradients, and achieves higher accuracy than other activation functions, such as sigmoid. The function expression of ELU is:

(2) $$f(x)=\{\begin{array}{c} \begin{array}{ccc} & x, & \end{array}x>0\\ \alpha \left(e^{x}-1\right),x\leq 0 \end{array}$$

### 2.4 Shared-weight-based multihead cross attention module

**Figure 2.**
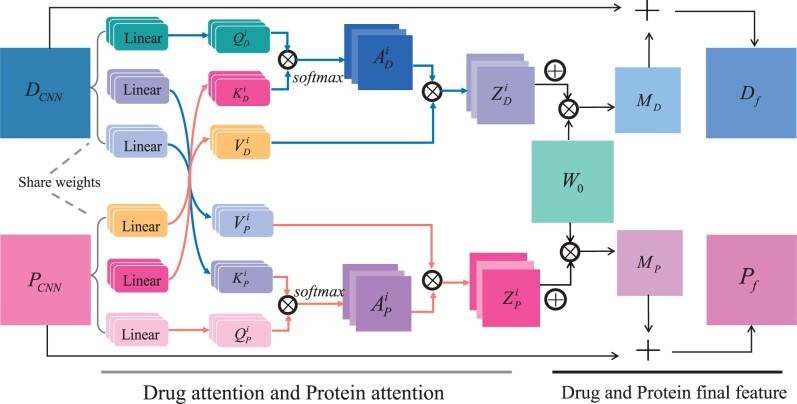
Diagram of the shared-weight-based multihead cross attention module. The queries, keys, and values of drugs and proteins are first computed through the weight-sharing linear layer. Then, the keys and values of drugs and proteins are swapped to compute the attention features $M_{D}$, $M_{P}\;$ of drugs and proteins through the multihead cross attention mechanism. Finally, the attention features are summed with the original features to get the final feature matrix $D_{f}$, $P_{f}$.

After the CNN block, we can obtain the drug feature matrix $D_{\mathrm{CNN}}$ and the protein feature matrix $P_{\mathrm{CNN}}$. The multihead cross attention module consists of the query vectors Q, the key vectors K, and the value vectors V and contains three steps. First, the feature matrix is passed through linear layers to compute Q, K, and V. In this step, the module is based on weight sharing. Second, a dot product operation is performed on Q and K, followed by softmax to obtain the normalized attention weight matrix A, then, A is multiplied with V to obtain the attention feature Z. Third, Z compute a weighted sum with $W_{0}$ to deduce the final attention feature matrix M, then, M is summed with the original feature to obtain the final feature matrix. Figure 2 shows the shared-weight-based multihead cross attention module. It contains two parts: (i) drug attention and protein attention and (ii) drug and protein final feature.

#### 2.4.1 Drug attention and protein attention

First, for drug attention, $D_{\mathrm{CNN}}\in R^{d_{\mathrm{embD}}\times d_{\mathrm{CNN}}}$ is passed through a linear layer to compute the query vectors of the drug $Q_{D}^{i}\in R^{d_{\mathrm{embD}}\times d_{h}}$, then, $P_{\mathrm{CNN}}\in R^{d_{\mathrm{embP}}\times d_{\mathrm{CNN}}}$ is passed through the other two linear layers to acquire the key vectors $K_{D}^{i}\in R^{d_{\mathrm{embP}}\times d_{h}}$ and the value vectors $V_{D}^{i}\in R^{d_{\mathrm{embP}}\times d_{h}}$ of the drug. Here, $d_{\mathrm{embD}}$ and $d_{\mathrm{embP}}$ are the drug and protein embedding dimensionality, $d_{\mathrm{CNN}}$ is the convolution layer dimensionality, h is the number of attention heads and $d_{h}=d_{\mathrm{CNN}}/h$ is the channel dimension. The three vectors of the drug are computed by the following equations:

(3) {QDi=DCNN×WQiKDi=PCNN×WKiVDi=PCNN×WVi i=1,2,…,h 

where $W_{Q}^{i},W_{K}^{i},W_{V}^{i}\in R^{d_{\mathrm{CNN}}\times d_{h}}$ are the learnable weight matrices in the three linear layers.

At the same time, the protein feature matrix $P_{\mathrm{CNN}}\in R^{d_{\mathrm{embP}}\times d_{\mathrm{CNN}}}$ obtained after convolution is passed through a linear layer to compute the protein query vectors $Q_{P}^{i}\in R^{d_{\mathrm{embP}}\times d_{h}}$. Subsequently, the drug feature matrix $D_{\mathrm{CNN}}\in R^{d_{\mathrm{embD}}\times d_{\mathrm{CNN}}}$ is passed through two other linear layers to compute the protein key vectors $K_{P}^{i}\in R^{d_{\mathrm{embD}}\times d_{h}}$ and value vectors $V_{P}^{i}\in R^{d_{\mathrm{embD}}\times d_{h}}$, which are computed by the following equation:

(4) {QPi=PCNN×WQiKPi=DCNN×WKiVPi=DCNN×WVi i=1,2,…,h 

where $W_{Q}^{i},W_{K}^{i},W_{V}^{i}\in R^{d_{\mathrm{CNN}}\times d_{h}}$ are the same weight matrices of the drug.

Second, a dot product operation is performed on $Q_{{\;}_{D}}^{i}$ and $K_{{\;}_{D}}^{i}$, followed by softmax to obtain the normalized drug attention weight matrices $A_{{\;}_{D}}^{i}\in R^{d_{\mathrm{embD}}\times d_{\mathrm{embP}}}$. The normalized protein attention weight matrices $A_{{\;}_{P}}^{i}\in R^{d_{\mathrm{embP}}\times d_{\mathrm{embD}}}$ are obtained by the same operation of $Q_{P}^{i}$ and $K_{P}^{i}$. Then, the attention weight metrics $A_{D}^{i}$ and $A_{{\;}_{P}}^{i}$ of each head are multiplied with the value matrix $V_{D}^{i}$ and $V_{{\;}_{P}}^{i}$ to obtain each of the attention features $Z_{D}^{i}\in R^{d_{\mathrm{embD}}\times d_{h}}$ and $Z_{P}^{i}\in R^{d_{\mathrm{embP}}\times d_{h}}$, which are computed by the following equation:

(5) $$Z_{D}^{i}=\mathrm{softmax}\left(\frac{Q_{D}^{i}K_{D}^{i\prime }}{\sqrt{d_{k_{D}^{i}}}}\right)V_{D}^{i}=A_{D}^{i}V_{D}^{i}$$

 

(6) $$Z_{P}^{i}=\mathrm{softmax}\left(\frac{Q_{P}^{i}K_{P}^{i\prime }}{\sqrt{d_{k_{P}^{i}}}}\right)V_{P}^{i}=A_{P}^{i}V_{P}^{i}$$

where $d_{k_{D}^{i}},d_{k_{P}^{i}}$ are the dimensions of the queries and keys.

#### 2.4.2 Drug and protein final feature

Third, the features of all the attention heads are concatenated along the channel dimensions and compute a weighted sum with $W_{0}\in R^{d_{h}\times d_{\mathrm{CNN}}}$ to deduce the final attention feature matrix $M_{D}\in R^{d_{\mathrm{embD}}\times d_{\mathrm{CNN}}}$ and $M_{P}\in R^{d_{\mathrm{embP}}\times d_{\mathrm{CNN}}}$, then, the attention features are summed with the original features to obtain the final feature matrices $D_{f}\in R^{d_{\mathrm{embD}}\times d_{\mathrm{CNN}}}$ and $P_{f}\in R^{d_{\mathrm{embP}}\times d_{\mathrm{CNN}}}$, which are updated by the following equations:

(7) $$\{\begin{array}{c} M_{D}=\mathrm{concat}\left(Z_{D}^{1},Z_{D}^{2},\ldots ,Z_{D}^{h}\right)\times W_{0}\\ M_{P}=\mathrm{concat}\left(Z_{P}^{1},Z_{P}^{2},\ldots ,Z_{P}^{h}\right)\times W_{0} \end{array}$$

 

(8) $$\{\begin{array}{c} D_{f}=0.5*M_{D}+0.5*D_{\mathrm{CNN}}\\ P_{f}=0.5*M_{P}+0.5*P_{\mathrm{CNN}} \end{array}$$

where $W_{0}$ is the learnable weight matrix.

The final feature matrix includes not only the original feature matrix information but also that obtained after cross attention. Thus, it does not result in the loss of information on the original features, and can better represent the fragment characteristics of drugs and proteins.

### 2.5 Predict interaction module

**Figure 3.**
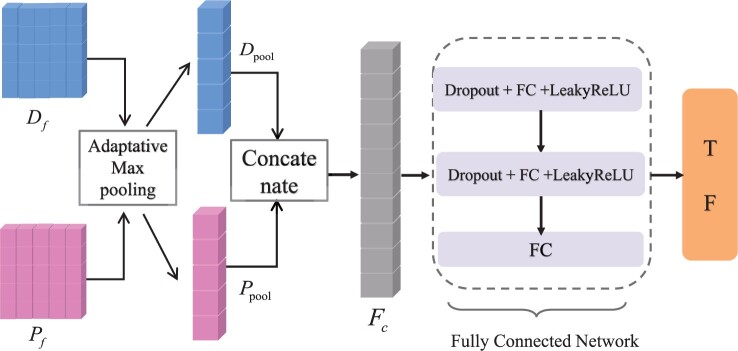
Interaction prediction. The 2D feature matrices are first downsampled into 1D feature maps by adaptive maximum pooling, and then, they are concatenated and fed into the FCN layer for classification prediction.

As shown in Fig. 3, the prediction module contains two adaptive maximum pooling layers, a concatenate layer and a FCN layer. The adaptive maximum pooling layers downsample the drug feature matrix $D_{f}\in R^{d_{\mathrm{embD}}\times d_{\mathrm{CNN}}}$ and the protein feature matrix $P_{f}\in R^{d_{\mathrm{embP}}\times d_{\mathrm{CNN}}}$ to generate 1D feature vectors $D_{\mathrm{pool}}\in R^{d_{\mathrm{CNN}}}$ and $P_{\mathrm{pool}}\in R^{d_{\mathrm{CNN}}}$:

(9) $$\{\begin{array}{c} D_{\mathrm{pool}}=\mathrm{AdaptiveMaxpool}\left(D_{f}\right)\\ P_{\mathrm{pool}}=\mathrm{AdaptiveMaxpool}\left(P_{f}\right) \end{array}$$

Next, connecting the drug and protein features results in $F_{c}\in R^{2d_{\mathrm{CNN}}\times 1}$:

(10) $$F_{c}=\mathrm{Concat}(D_{\mathrm{pool}},P_{\mathrm{pool}})$$

Finally, DTI prediction is performed through a FCN layer. It consists of three parts: a dropout layer, a linear layer, and a leaky ReLU activation function layer (Maas *et al.* 2013). The first two layers include the dropout layer, the linear layer, and the leaky ReLU activation layer, while the last layer only consists of the linear layer.

### 2.6 Loss function

The loss function is indispensable in the model training process of deep learning as it can measure the difference between the model predicted value and the real value. The process of training the model is optimizing the loss function: the smaller the value of the loss function is, the better the model fits the sample. We choose binary cross-entropy loss (BCELoss) as the loss function, for it possesses the characteristics of easy optimization and strong interpretability and can improve the stability of numerical computation:

(11) $$\mathrm{loss}=\frac{1}{N}\sum_{i=1}^{N}-\left(y_{i}\;\text{log}\;\left(p_{i}\right)+\left(1-y_{i}\right)\;\text{log}\;\left(p_{i}\right)\right)$$

where N represents the total number of samples trained, p represents the predicted probability, and $y_{i}$ represents the true label value.

## 3 Experiments

### 3.1 Baselines

We compare FMCA-DTI with the following four baselines on DTI prediction.

#### 3.1.1 GNN-CPI

GNN-CPI (Tsubaki *et al.* 2019) uses 1D-CNN and graph neural network (GNN) to encode proteins and drugs. The graph structure of a compound molecule is encoded as a fixed-size vector using a GNN. 1D-CNN was used to encode the amino acid chains of proteins into the same embedding space as the GNN output. After that, a one-sided attention mechanism is applied to extract protein features. Finally, the feature vectors are fed into the fully connected neural network for predicting interaction via a concatenation operation.

#### 3.1.2 GNN-PT

GNN-PT (Wang et al. 2020) encodes drugs by GNN and uses a transformer and CNN to represent proteins features. The one-side attention mechanism is also used to give high attention values to the protein sequences essential for the drug, and finally, protein feature vectors are obtained. Finally, the values are input to the fully connected layer for prediction via concatenate operations.

#### 3.1.3 DeepEmbedding-DTI

DeepEmbedding-DTI (Chen *et al.* 2021) utilizes a bidirectional encoder representation (BERT) approach to extract substructural features from protein sequences, and implements local breadth-first search to learn subgraph information from molecular graphs. The drugs and proteins features are encoded by GNN and a bidirectional LSTM with an attention mechanism. Finally, prediction is made by concatenate operations fed to the fully connected layer.

#### 3.1.4 BCM-DTI

For a given drug–target pair in BCM-DTI (Dou *et al.* 2023), the drug SMILES and protein amino acid sequences are first segmented and extracted in fragments using BCM and CFM. The initial coding layer is, then, utilized to encode the different fragment types extracted to obtain the embedding vectors. Next, two parallel CNNs are utilized to learn the features of these fragments. Finally, the fully connected layer is applied to predict DTIs.

### 3.2 Datasets

We used three public datasets, including BioSNAP (Zitnik *et al.* 2018), Human (Liu *et al.* 2015), and Celegans (Tsubaki *et al.* 2019) to train and evaluate our model. The BioSNAP dataset consists of 4510 drugs and 2181 proteins. The Human and Celegans datasets are fully cover the human protein kinome and are balanced datasets of positive and negative samples included in the entire kinome (Liu *et al.* 2015). (The datasets in our training process are introduced in Supplementary Material S1.) The drugs, proteins and the number of positive and negative samples included in the different datasets are shown in Table 1.

**Table 1.** Summary of the three benchmark datasets.

Datasets	Drug	Protein	Interaction	Positive	Negative
BioSNAP	4510	2181	27 464	13 830	13 634
Human	2726	2001	6728	3364	3364
Celegans	1767	1876	7786	3893	3893

### 3.3 Hyper-parameter settings

The learning rate, the batch size, the weight decay coefficient and the dropout rate are determined by grid-search on the BioSNAP dataset (see Supplementary Material S2 for calculation details). In general, the optimized learning rate, batch size, weight decay coefficient and dropout rate are 1e-5, 64, 1e-4, and 0.1, respectively. In the CNN blocks, we set the kernel sizes of three convolutions are both 3 for drugs, while for proteins, the kernel sizes of three convolutions are set to 4, 6, and 8. The number of fully connected layers for the prediction module is 3. The heads of attention is 4 and the embedding layer dimension is 512. (The experiment details are presented in Supplementary Material S3.)

### 3.4 Evaluation strategies

We set different seeds and the dataset is divided into the training dataset and the test dataset according to the ratio of 8:2. Then, for the training dataset, we apply the 5-fold cross-validation method to train the model. That is, the training dataset is again divided into five parts, four of the folds as the final training dataset to train the model, and the remaining fold data as the validation dataset to validate the model. We use the area under the operating characteristic curve (AUC), the area under the precision–recall curve (AUPR), accuracy, precision, and recall as metrics to measure the binary classification performance of the model. (The detailed information of evaluation criteria can be found in Supplementary Material S4.)

## 4 Results

### 4.1 Performance analysis

To evaluate the performance of our proposed FMCA-DTI against baselines, we select three datasets for training: BioSNAP, Human, and Celegans, and evaluate the models with the five metrics introduced above. During the training process, the hyper-parameters of the different models are set to the same parameters as in the original paper to ensure the fairness of the assessment (see details in Supplementary Material S5).

**Figure 4.**
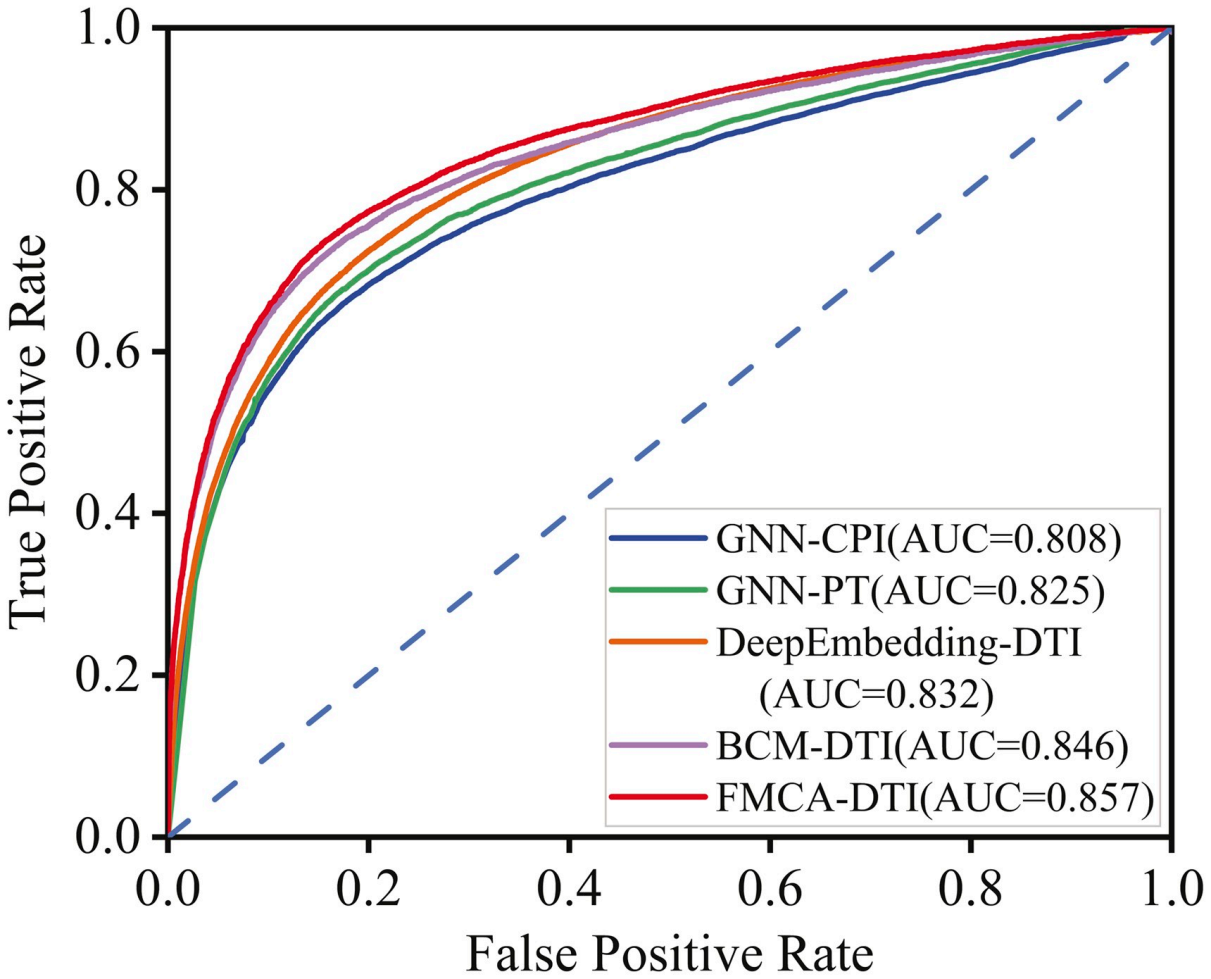
The ROC curves illustrate the results of our method compared with four baselines on the BioSNAP datasets.

First, on the BioSNAP dataset, we conduct comparative experiments, with Table 2 showing the performance results of AUC, AUPR, accuracy, precision, and recall of different baselines and FMCA-DTI. As is summarized in Table 2, our method yields better results than all previously mentioned state-of-the-art models. Specifically, AUC is improved by 1.1% and AUPR is improved by 0.8% when compared with BCM-DTI, accuracy is improved by 2.9% when compared with GNN-PT, precision rate is improved by 6.4% when compared with DeepEmbedding-DTI. We visualized the ROC curve results of the comparison experiments on BioSNAP datasets, as shown in Fig. 4.

**Table 2.** Comparative results on the BioSNAP dataset **(Best).**

Datasets	Methods	AUC	AUPR	Accuracy	Precision	Recall
BioSNAP	GNN-CPI	0.808	0.810	0.735	0.705	0.675
	GNN-PT	0.825	0.832	0.758	0.789	0.703
	DeepEmbedding-DTI	0.832	0.841	0.757	0.764	0.705
	BCM-DTI	0.846	0.864	0.781	0.820	0.720
	FMCA-DTI	**0.857**	**0.872**	**0.787**	**0.828**	**0.727**

Second, Table 3 shows the results of the comparison experiments on the Human dataset. From the results, we can conclude that compared with the best metrics, our method improves by 0.5% in the AUC metric, 1.1% in accuracy, 0.5% in the precision metric, and 1.7% in the recall metric.

**Table 3.** Comparative results on the Human dataset **(Best).**

Datasets	Methods	AUC	AUPR	Accuracy	Precision	Recall
Human	GNN-CPI	0.964	0.879	0.880	0.869	0.872
	GNN-PT	0.967	0.916	0.883	0.908	0.862
	DeepEmbedding-DTI	0.971	0.930	0.885	0.917	0.904
	BCM-DTI	0.987	0.988	0.939	0.963	0.909
	FMCA-DTI	**0.992**	**0.991**	**0.950**	**0.968**	**0.926**

Finally, we train and evaluate our method and baselines on the Celegans dataset, with the evaluation results shown in Table 4. From the experimental results, we can conclude that our method is located in the first place and passes through the BCM-DTI with an advantage of 0.6%, 0.5%, and 2.6% in the metrics of AUC, AUPR, and accuracy.

**Table 4.** Comparative results on the Celegans dataset **(Best).**

Datasets	Methods	AUC	AUPR	Accuracy	Precision	Recall
Celegans	GNN-CPI	0.949	0.761	0.915	0.952	0.968
	GNN-PT	0.975	0.928	0.930	0.972	0.921
	DeepEmbedding-DTI	0.983	0.920	0.944	0.977	0.956
	BCM-DTI	0.990	0.991	0.949	0.961	0.935
	FMCA-DTI	**0.996**	**0.996**	**0.975**	**0.992**	**0.976**

### 4.2 Ablation experiments

**Figure 5.**
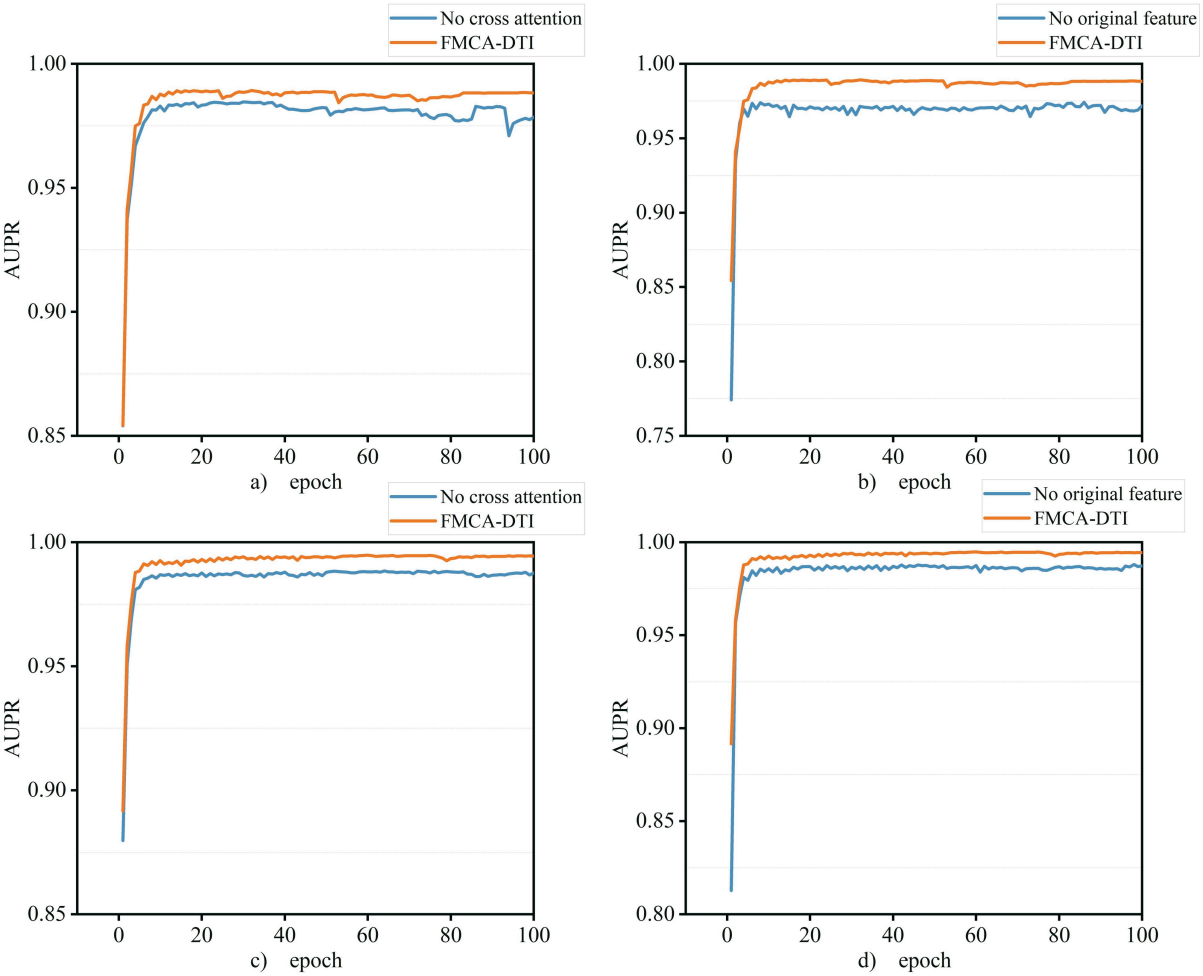
Ablation experiments results on AUPR. (a) and (b) show the comparison on the Human dataset. (c) and (d) show the comparison on the Celegans dataset. No-cross attention indicates the model with the multihead cross attention module removed and no-original feature indicates the model without the original feature matrix.

We conduct ablation experiments on the Human dataset and the Celegans dataset, using AUC, AUPR, precision, and recall as evaluation metrics to assess the effectiveness of the multihead cross attention mechanism as well as the importance of the original feature information. The results presented in Table 5 and the AUPR results on the Human data and Celegans dataset are shown in Fig. 5.

**Table 5.** Ablation experiments on the Human and Celegans datasets **(Best).**

Datasets	Methods	AUC	AUPR	Precision	Recall
Human	No-cross attention	0.985	0.984	0.952	0.910
	No-original feature	0.965	0.967	0.922	0.909
	FMCA-DTI	**0.992**	**0.991**	**0.968**	**0.926**
Celegans	No-cross attention	0.994	0.994	0.967	0.958
	No-original feature	0.985	0.985	0.957	0.938
	FMCA-DTI	**0.996**	**0.996**	**0.992**	**0.976**

We first remove the multihead cross attention module to construct a variant model, named no-cross attention. We compare the variant model with FMCA-DTI to verify the effectiveness of the multihead cross attention mechanism. Followingly, we construct another variant model by not using the original features but only the full attention feature matrix as the final feature matrix and name it the no-original feature. We further prove the importance of the original features by comparing the variant no-original feature with our model.

As shown in Table 5, FMCA-DTI is in an advantageous position in all four metrics on the two datasets when compared to the first variant model. The multihead cross attention module improves the AUC by 0.7% and 0.2%, the AUPR by 0.7% and 0.2%, the precision by 1.6% and 2.5%, and the recall by 1.6% and 1.8%. The importance of the original feature matrix for improving model performance is demonstrated when compared to the second variant model. As shown in Table 5 and Fig. 5b and d, AUC, AUPR, precision, and recall are improved by 2.7%, 2.4%, 4.6%, and 1.7% on the Human dataset, and 1.1%, 1.1%, 3.5%, and 3.8% on the Celegans dataset. The results demonstrate that the multihead cross attention module and original features can effectively improve the model performance.

### 4.3 Case study

To evaluate the reliability of our model, we analyzed the accuracy of DTI prediction results of some drugs and proteins from the BioSNAP dataset. First, we randomly selected two drugs (Ropinirole and Tretinoin) with no less than ten related proteins and analyzed the accuracy of predicting the drugs. The results are shown in Table 6, listing the relevant proteins predicted by our model for the two drugs, the ground-truth results, and the prediction. Specifically, the accuracy for Ropinirole and Tretinoin can achieve more than 90%, with only one false prediction.

**Table 6.** Prediction results of drugs Ropinirole and Tretinoin.

Drug	Protein	True label	Predict label
DB00268-Ropinirole	P41231	False	False
	P08913	True	True
	P41595	True	True
	P21917	True	True
	Q9UQL6	False	True
	P28222	True	True
	P10635	True	True
	P05177	True	True
	P18089	True	True
	P35462	True	True
	P21728	True	True
Accuracy			90.9%
DB00755-Tretinoin	P48443	True	True
	P13631	True	False
	P00352	True	True
	P11712	True	True
	P04798	True	True
	P08684	True	True
	P24462	True	True
	P33260	True	True
	P10632	True	True
	P28702	True	True
Accuracy			90%

Second, we randomly selected two proteins (UGT2B7 and MAOA), chose the drugs that interact with them for testing, and analyzed the accuracy of the prediction. The predictions are shown in Table 7. Regarding the two proteins, our model can achieve an accuracy of over 90%, with only one false prediction respectively in drugs DB01219 and DB00918.

**Table 7.** Prediction results of target proteins UGT2B7 and MAOA.

Protein	Drug	True label	Predict label
P16662-UGT2B7	DB01252	True	True
	DB06777	True	True
	DB00870	True	True
	DB01219	False	True
	DB00295	True	True
	DB07625	False	False
	DB01024	True	True
	DB01227	False	False
	DB00749	True	True
	DB06292	True	True
	DB00712	True	True
Accuracy			90.9%
P21397-MAOA	DB00721	True	True
	DB07641	True	True
	DB00918	True	False
	DB01472	True	True
	DB00752	True	True
	DB00191	True	True
	DB00988	True	True
	DB00953	True	True
	DB13946	True	True
	DB04880	False	False
Accuracy			90%

## 5 Conclusion

In this article, we propose an end-to-end deep learning model, known as FMCA-DTI, which is a fragment-oriented approach based on a multihead cross attention mechanism for predicting DTIs. The model segments drug molecules and protein amino acid sequences into different fragment types separately, then, extracts features from these fragments by CNNS, and finally utilizes the multihead cross attention mechanism and FCN for DTI prediction. This model considers multiple fragments of drugs and proteins, and obtains the fragment types of drugs and proteins via BCM and CFM, effectively extracting robust interaction features between different fragments of drugs and proteins using the shared-weight-based multihead cross attention mechanism. To validate the effectiveness of the model, we conducted comparative experiments with the best four baselines on three public datasets, and the results show that our methods significantly improve prediction performance. In addition, we also conducted ablation experiments on two datasets, and the results further confirmed the effectiveness of our proposed model. In future work, we will select more datasets with balanced positive and negative samples for experiments and apply various attention mechanisms to improve the prediction performance of the model.

## Supplementary Material

btae347_Supplementary_Data
