# Supplementary material for: FMCA-DTI: a fragment-oriented method based on a multihead cross attention mechanism to improve drug–target interaction prediction
Source: Bioinformatics. 2024 May 29;40(6):btae347. doi: 10.1093/bioinformatics/btae347 (PMC11256963; doi:10.1093/bioinformatics/btae347)
Supplement: btae347_Supplementary_Data [file btae347_supplementary_data.zip › Supplementary Material.docx]

# Supplementary Material

**FMCA-DTI: A Fragment-oriented method based on a Multihead Cross Attention mechanism to improve Drug-Target Interaction prediction**

Qi Zhang^[[1]](#footnote-0)^, Le Zuo^1^, Ying Ren^1^, Siyuan Wang^1^, Wenfa Wang^1^, Lerong Ma^1^, Jing Zhang^[[2]](#footnote-1),^^[[3]](#footnote-2),^^[[4]](#footnote-3)^* and Bisheng Xia^1,*^

1. **Dataset** **inforamtion**

**BioSNAP dataset:** The BioSNAP dataset is created from the DrugBank database (Wishart *et al.* 2008) by Huang et al. (Huang *et al.* 2021) and Zitnik et al. (Zitnik *et al.* 2018), It is a balanced dataset with validated positive interaction and an equal number of negative samples randomly obtained from unseen pairs. The negative pairs sample from the invisible pairs via the methods mentioned in (Zitnik *et al.* 2018, Zhang and Chen 2018), eventually acquiring a new balanced dataset with the same positive and negative sample cases.

**Human and Celegans datasets:** Both are fully cover the human protein kinome and are balanced datasets of positive and negative samples included in the entire kinome. The kinome consists of known and clinical kinase inhibitors (Liu *et al.* 2015). The positive samples are from the DrugBank database (Wishart *et al.* 2008) and the Matador database (Günther *et al.* 2008), and negative samples are screened by a systematic approach under the assumption that similar compounds bind to similar target proteins (Liu *et al.* 2015).

1. **FMCA-DTI implementation**

The learning rate, the batch size, the weight decay coefficient and the dropout rate are determined by grid-search on the BioSNAP dataset. In grid-search, we first determine the learning rate, which is in [1e-2, 1e-3, 1e-4, 1e-5, 1e-6] and the batch size, which is in [16, 32, 64, 128, 256, 512]. After these two hyper-parameters are fixed, we select the weight decay coefficient and dropout rate, which are in [1e-2, 1e-3, 1e-4, 1e-5, 1e-6] and [0.1, 0.2, 0.3, 0.4, 0.5].

For parameter optimization, we use the AdamW optimizer (Kingma and Ba 2014) to update the network weights. The number of training rounds is set to 100, with an early stopping strategy to avoid overfitting. The model is analyzed using PyTorch (Paszke *et al.* 2019), for training, we used the following server configuration: GPU: RTX A5000, 24 GB, CPU: Intel(R) Xeon(R) Gold 6330, 30 GB.

## **Parameters analysis**

To further determine the size of the different heads of attention and the embedding layer dimensions, we perform two experiments on the BioSNAP dataset.

Firstly, we test the influence of different heads of attention in the cross attention module. We perform the experiment on the BioSNAP dataset where the number of attention heads vary in 1, 2, 4, 8, and 16. We obtain that when the number of attention heads is 4, the AUC and AUPR are at their peak, as depicted in FigureS 1. The results show that different attention heads can influence the performance of the model.

FigureS 1 The AUC and AUPR results of our method with different attention heads on the BioSNAP datasets.

Next, we test the influence of the embedding layer dimensions. We perform the another experiment on the BioSNAP dataset where the embedding layer dimensions vary in 64, 128, 256, 512, 1024. We obtain that when the embedding layer dimensions is 512, the AUC and AUPR are at their peak, as depicted in FigureS 2. The results show that different embedding layer dimensions can influence the performance of the model.

FigureS 2 The AUC and AUPR results of our method with different embedding layer dimension on the BioSNAP datasets.

1. **Evaluation metrics**

We use the area under the operating characteristic curve (AUC), the area under the precision-recall curve (AUPR), accuracy, precision, and recall as metrics to measure the binary classification performance of the model. The best results for each metric are bolded. Precision is the proportion of true positive cases among all samples predicted as positive by the model, which measures the model’s ability to reliably predict positive cases. Recall is the proportion of all true positive examples correctly predicted as positive examples by the model and can assess the ability to recognize positive examples. Their formulas are as follows:

$precision=\frac{TP}{TP+FP}\text{，}recall=\frac{TP}{TP+FN}$ (1)

where $TP$ refers to the number of true positive samples, $FP$ refers to the number of false positive samples, and $FN$ refers to the number of false negative samples. The receiver operating characteristic curve (ROC) is a representation of the relationship between the true positive rate (TPR) and false positive rate (FPR), indicating the performance of the classifier under different thresholds,

$FPR=\frac{FP}{TN+FP}\text{，}TPR=\frac{TP}{TP+FN}$ (2)

On the ROC curve, the horizontal axis represents the false positive rate and the vertical axis represents the true positive rate. AUC is the area under the ROC, which represents the comprehensive performance of the model’s classification ability over the entire threshold range. The closer the AUC is to 1, the better the model performance. AUPR is the area under the precision-recall curve.

1. **Hyper-parameters analysis on the BioSNAP dataset**

In order to select the learning rate, the batch size, the weight decay coefficient, and the dropout rate for the different models, we perform the experiment comparing these hyper-parameters of the FMCA-DTI model with those used in the baselines in the original paper on the BioSNAP dataset. We use the area under the operating characteristic curve (AUC), the area under the precision-recall curve (AUPR), accuracy as metrics to measure the performance. The results are shown in Table 1.

**Table 1** Comparative results on the BioSNAP dataset with different hyper-parameters

| Model | Hyper-parameters | AUC | AUPR | Accuracy |
| --- | --- | --- | --- | --- |
| GNN-CPI | Original | **0.808** | **0.810** | **0.735** |
|  | Same with FMCA-DTI | 0.775 | 0.809 | 0.694 |
| GNN-PT | Original | **0.825** | **0.832** | **0.758** |
|  | Same with FMCA-DTI | 0.785 | 0.819 | 0.724 |
| DeepEmbedding-DTI | Original | **0.832** | **0.841** | **0.757** |
|  | Same with FMCA-DTI | 0.807 | 0.835 | 0.730 |
| BCM-DTI | Original | **0.846** | **0.864** | **0.781** |
|  | Same with FMCA-DTI | 0.818 | 0.841 | 0.768 |
| FMCA-DTI |  | **0.857** | **0.872** | **0.787** |

**References**

Günther S *et al.* (2008) SuperTarget and Matador: resources for exploring drug-target relationships. *Nucleic Acids Res*, **36**, D919-22.

Huang K *et al.* (2021) MolTrans: Molecular Interaction Transformer for drug-target interaction prediction. *Bioinformatics*, **37**, 830-6.

Kingma DP and Ba J. (2014) Adam: A Method for Stochastic Optimization. arXiv, arXiv:1412.6980, preprint: not peer reviewed.

Liu H *et al.* (2015) Improving compound-protein interaction prediction by building up highly credible negative samples. *Bioinformatics*, **31**, i221-9.

Paszke A *et al.* (2019) PyTorch: An Imperative Style, High-Performance Deep Learning Library. arXiv, arXiv:1912.01703, preprint: not peer reviewed.

Wishart DS *et al.* (2008) DrugBank: a knowledgebase for drugs, drug actions and drug targets. *Nucleic Acids Res*, **36**, D901-6.

Zhang M and Chen Y. (2018) Link prediction based on graph neural networks. In: *Proceedings of the 32nd International Conference on Neural Information Processing Systems*, Montréal, Canada. 5171–81.

Zitnik M *et al.* (2018) BioSNAP Datasets: Stanford Biomedical Network Dataset Collection.

1. College of Mathematics and Computer Science, Yan' an University, Yan' an 716000, China [↑](#footnote-ref-0)
2. Medical College of Yan' an University, Yan' an University, Yan' an 716000, China; [↑](#footnote-ref-1)
3. Medical research and experimental center, The Second Affiliated Hospital of Xi 'an Medical University, China. [↑](#footnote-ref-2)
4. *Corresponding author. Medical College of Yan' an University, Yan' an University, Yan' an 716000, China. Medical research and experimental center, The Second Affiliated Hospital of Xi 'an Medical University, China. E-mail: yadxzj@163.com; College of Mathematics and Computer Science, Yan' an University, Yan' an 716000, China. E-mail: bishengxia@163.com [↑](#footnote-ref-3)
